# Supplementary material for: Novel Salinity-Tolerant Third-Generation Hybrid Rice Developed via CRISPR/Cas9-Mediated Gene Editing
Source: Int J Mol Sci. 2023 Apr 28;24(9):8025. doi: 10.3390/ijms24098025 (PMC10179023; doi:10.3390/ijms24098025)
Supplement: Supplementary file 1 [file ijms-24-08025-s001.zip › ijms-2276414-supplementary.pdf]

Supplementary Materials

Supplementary Table S1. Primers used in this study.

Primer Name	Base Sequence (5′→3′)	Purpose
22KT1-F	GGCACATGGCGCGCACGCACGTA	Construction of 22KT1 target site
22KT1-R	AAACTACGTGCGTGCGCGCCATG	Construction of 22KT1 target site
22KT2-F	GCCGCGCCATGTCGTCCGCCGCT	Construction of 22KT2 target site
22KT1-R	GGCACATGGCGCGCACGCACGTA	Construction of 22KT2 target site
22T3-F	GGCACACGTACCACCAAATGCTA	Construction of 22T3 target site
22T3-R	AAACTAGCATTTGGTGGTACGTG	Construction of 22T3 target site
U-F	AAACTACGTGCGTGCGCGCCATG	U#-T-gRNA expression-box frist PCR
gRNA-R	GCCGCGCCATGTCGTCCGCCGCT	U#-T-gRNA expression-box frist PCR
B1′	TTCAGAGGTCTCTCTCGCACTGGAA TCGGCAGCAAAGG	U#-T-gRNA expression-box second PCR
B2	AGCGTGGGTCTCGTCAGGGTCCATC CACTCCAAGCTC	U#-T-gRNA expression-box second PCR
B2′	TTCAGAGGTCTCTCTGACACTGGAA TCGGCAGCAAAGG	U#-T-gRNA expression-box second PCR
BL	AGCGTGGGTCTCGACCGGGTCCATC CACTCCAAGCTC	U#-T-gRNA expression-box second PCR
SP1	CCCGACATAGATGCAATAACTTC	CRISPR/Cas9 expression-vector verification
SP2	GCGCGGTGTCATCTATGTTACT	CRISPR/Cas9 expression-vector verification
HPT-F1	CTTCTGCGGGCGATTTGT	Transgenic plant detection
HPT-R1	CAGCGTCTCCGACCTGAT	Transgenic plant detection
Cas9-F1	GTTGGTATTCACGGGGTGCCT	Transgenic plant detection
Cas9-R1	CGACGATGTTGCCGAAGATGG	Transgenic plant detection
RR-CJ3F	GGATCAGCTGTTGTGTAAAAATGTC	22KT12,22T3 target mutation detection
RR-CJ3R	CTTGCAACATTTTCCCTGGTGAG	22KT12,22T3 target mutation detection

Supplementary Table S2. CRISPR/Cas9-induced mutations in *OsRR22* and Transgene-free ratio in T1 generation.

T0 Plant	Target Site	Host Cultivar	No. of Plants Examined	Transgene-Free Ratio (%) in T1
T1430	22KT12	733B	18	33.33 (6/18)
T1431	22KT12	733B	18	27.78 (5/18)
T1432	22KT12	733B	18	0 (0/18)
T1447	22KT12	733B	18	16.67 (3/18)
T1448	22KT12	733B	18	22.23 (4/18)
T1347	22T3	HZ	18	11.12 (2/18)
T1348	22T3	HZ	18	0 (0/18)
T1349	22T3	HZ	18	27.78 (5/18)
T1350	22T3	HZ	18	27.78 (5/18)
T1351	22T3	HZ	18	16.67 (3/18)

Supplementary Table S3. Mutation detections in the putative CRISPR/Cas9 off-target sites.

Target Site	Putative Off-Target Site	Putative Off-Target Locus	Sequence of Putative Off-Target Site [1]	No. of Mismatching Bases	No. of Plants Detected	No. of Plants with Mutations
22KT1	off-22KT1-1	Chr11:1220469-1220488	TGATTAAAAGAACCACAGAA AGG	4	90	0
22KT1	off-22KT1-2	Chr11:14999083-14999102	TTCTTAGAAGAATCACAGGT AGG	4	90	0
22KT1	off-22KT1-3	Chr11:4021308-4021327	TGCTCAGAAGATCCACAGTT AGG	3	90	0
22KT1	off-22KT1-4	Chr11:8539864-8539883	TTAGCAGCAGCACCACAGGT TGG	4	90	0
22KT2	off-22KT2-1	Chr3:35790995-35791014	CTGGGCTCTTATGCAGCAGA TGG	4	90	0
22KT2	off-22KT2-2	Chr3:12627682-12627701	CCTCTCTTCTTTGCAGCTGA TGG	4	90	0
22T3	off-22T3-1	Chr7:22781210-22781229	TGACAATGAATCTGATGAAG AGG	4	90	0
22T3	off-22T3-2	Chr1:33054780-33054799	TGACCAAGGACGTGATGAAG GGG	5	90	0
22T3	off-22T3-3	Chr1:32373735-32373754	AGTCAAAGACTGTCAGGAAA GGG	4	90	0
22T3	off-22T3-4	Chr1:32373735-32373754	AGACCAAAACTGTATTGAAG GGG	4	90	0

[1] The base that matched the sgRNA are marked in black. The base that mismatched the sgRNA are marked in red. The sequences of the PAM are underlined.
